# Sarcopenia, Exercise and Quality of Life

**DOI:** 10.3390/ijerph18105156

**Published:** 2021-05-13

**Authors:** Juan Mielgo-Ayuso, Diego Fernández-Lázaro

**Affiliations:** 1 Department of Health Sciences, Faculty of Health Sciences, University of Burgos, 09001 Burgos, Spain; 2 Department of Cellular Biology, Histology and Pharmacology, Faculty of Health Sciences, University of Valladolid, Campus of Soria, 42003 Soria, Spain; diego.fernandez.lazaro@uva.es; 3 Neurobiology Research Group, Faculty of Medicine, University of Valladolid, 47002 Valladolid, Spain

The loss of strength, power, and muscle mass caused by the progressive deterioration from aging is known as “sarcopenia.” This age-related disease is closely related to the progressive loss of physical and cognitive abilities. The etiology of sarcopenia is multifactorial: hormonal, neurological, muscular, immunological, nutritional, or related to a sedentary lifestyle. These factors cause pathophysiological changes at the neuromuscular and tendon level, increased risk of chronic diseases (diabetes and osteoporosis), suppression of ketogenesis and changes in body temperature. Muscle mass gradually decreases by 3% to 8% every decade from the age of 30 onwards, and is particularly accentuated from 60 ahead. This results in a progressive decline in strength that contributes significantly to disability and loss of independence in the elderly. Therefore, to treat and delay sarcopenia, decisions regarding lifestyle habits must be taken into account. In addition, with the physiological and systemic changes in the body as age advances and accelerates the progression of this syndrome, lifestyle factors are much more controllable, and the inclusion of exercise (both endurance and strength) in a healthy lifestyle is of paramount importance. Therefore, in this Special Issue, “*Sarcopenia, Exercise and Quality of Life*” we focus on the current state of knowledge on the links between sarcopenia, exercise, and quality of life.

This special edition of the *Journal of Environmental Research and Public Health* (*IJERPH*) has brought together eleven research manuscripts [1,2,3,4,5,6,7,8,9,10,11] and a brief report [12]. This special issue, entitled “*Sarcopenia, Exercise and Quality of Life*” gathered 12 manuscripts [1,2,3,4,5,6,7,8,9,10,11,12]; four (33.3%) manuscripts are related to the impact of physical activity concerning sarcopenia [4,6,7,10]. Two manuscripts were used for the Wii Fit^®^ games as an intervention tool for physical activity in institutionalized elderly patients [4,6]. Wii Fit^®^ represents an innovative element for the practice of exercise, of low economic cost compared to other traditional programs, which allows working of all the components of exercise obtaining favorable results on the biomarkers of physiological evaluation of the patient. Gonzalez-Bernal et al. [4] demonstrated in institutionalized older adults that Wii Fit^®^ allows in eight weeks of physical practice through Wii Fit^®^ games a significant decrease in the risk of falls and a significant improvement in static balance.

Additionally, the improvements obtained in the patients’ physical condition allow us to affirm that the practice of exercise for the use of Wii Fit^®^ games is an adequate therapeutic strategy, minimally aggressive, and reduces the degree of disability and risk of hospitalization [4]. Another study [6] by the same research group, using a similar research methodology, managed to stimulate and generate emotions much more beneficial than the usual practice of exercise, allowing improvement of attention and memory processes, attenuating the negative impacts of depression, anxiety, and apathy. Therefore, the Wii Fit^®^ allows the reaching of adequate cognitive and psychological levels that directly influence performance improvement in basic and instrumental activities of daily living (ADL) [6].

On the other hand, Tous-Espelosín et al. [10] and Papadopoulou et al. [7] showed the protective role of exercise against obesity and other associated comorbidities in older adults. EXERDIET-HTA is a study [10] that performs high-intensity interval training (HIIT) interventions, plus a special diet as “Dietary Approaches to Stop Hypertension” (DASH) on physically inactive and obese study subjects with primary arterial hypertension (HTA) (*n* = 253) for 16 weeks compared to a healthy population sample (*n* = 30). DASH is a suitable regimen for people who suffer from high blood pressure or prehypertension because it tends to lower it without medication. The HIIT exercise plus DASH provided stimulated vitality in the patients with HTA after 16 weeks. Further, the EXERDIET-HTA intervention provided higher values in “physical functioning” and “general health.” Significantly, the low-volume HIIT intervention stimulated positive changes in social functioning and mental health. These authors affirm that HIIT interventions allow changes in quality of life, especially in the physical and psychological dimensions. This study used SF-36 as a subjective questionnaire to assess the quality of life outcomes [10]. In this line, the study of Papadopoulou et al. [7], carried out in 102 older people, 50 men and 52 women, aged 60–83 years, described that the practice of physical activity (2–3 days per week) allows for the control of bodyweight, and therefore minimizes the health risks of obesity, especially in older adults. This study highlights that non-pharmacological interventions based on exercise can attenuate the risk of obesity in the elderly.

In this special issue, three manuscripts (25%) [1,2,5] evaluated modern diagnostic methods of sarcopenia, with the aim of more accurately identifying this pathology. Cebria et al. [2] and Guillamon-Escudero et al. [5] used the European Working Group on Sarcopenia in Older People criteria established in 2019 (EWGSOP2). Both studies aimed to confirm the reliability of EGWSOP2 in populations of institutionalized older adults (*n* = 132) (5) and populations attending municipal activity centers for the autonomous elderly (*n* = 202) [2]. The target population is elderly residents in Valencia (Spain) [2,5]. Guillamon-Escudero et al. [5] consider the SARC-F questionnaire, included in EWGSOP2, to be the critical tool for diagnosing 40% of sarcopenia cases in their population. Cebria et al. [2] state that the Barthel Index (BI) and Abbreviated Charlson’s Comorbidity Index (ACCI) are the appropriate markers for early diagnosis of sarcopenia. Castillo-Olea et al. [1] use automated practices of clinical sarcopenia diagnosis on 166 elderly patients during six months, evaluating a total of 99 variables. The results of this study show that the new variables considered key by the authors (age, systolic blood pressure, number of chronic diseases and sodium) should be complemented with those classically used for sarcopenia diagnosis when assessing patients with moderate and severe sarcopenia. Overall, the new sarcopenia diagnostic techniques consider different predictors, which are essential depending on the particular characteristics of the study population. However, all of these show a high efficacy for the diagnosis of individuals with sarcopenia.

Sarcopenia is a medical term of Greek etymology, meaning scarcity of flesh (muscle), and refers to the loss of skeletal muscle mass during aging. This translates into an increased risk of poor quality of life, physical disability, frequent falls and weakness, and death. In this sense, four manuscripts [3,8,9,11] in this special issue, corresponding to 33.3% of the total, corroborate that muscle loss is the typical consequence of all older adults. The study by Patiño-Villada et al. [8] considers the combination of high muscle mass and shallow fat mass a highly healthy variety. The elderly presenting these characteristics obtained better results in muscle strength and functional capacity tests. These results, brought in from a sample of 143 non-institutionalized older subjects aged 70 years, estimate that appendicular lean mass (ALM)—the sum of lean mass in arms and legs excluding fat and bone mass using dual-energy X-ray absorptiometry (DXA)—is the body measure most closely related to muscle strength loss. This positions ALM as a priority clinical measure of sarcopenia over muscle mass or osteoporosis [8]. However, Myong-Wu et al. [9] reported muscle quality as the identifying marker of the sarcopenic patient. These authors, using sectional computed tomography scans on 59 elderly women (>65 years), indicate that muscle quality is the crucial element in the confirmatory diagnosis of an older adult patient with sarcopenia. However, muscle growth biomarkers (GDF-15, myostatin, activin A, and follistatin) were not effective in diagnosing sarcopenia. This suggests that diagnostic imaging techniques are more advantageous than molecular biology analytical determinations. Dopsaj et al. [3] evaluated muscle components by measuring different body composition variables. Among these variables body, skeletal muscle mass index (SMMI; kg/m^2^) and percentage of skeletal muscle mass (PSMM; %) are the most sensitive to age. SMMI is independent of sex, but PSMM is sex dependent. The optimal values of these muscle indicators do not seem sufficient for the older adult patient to have a non-pathogenic musculoskeletal status. Still, it is necessary to also consider body fat mass. Thus, these authors [3] consider body fat plus muscle mass assessment essential in the description of the sarcopenic patient. Also, muscle rehabilitation work of the lower limbs (quadriceps and hamstrings) is vital to increasing muscle mass. This results in improved balance and stability in older women (60–70 years old) with knee osteoarthritis (KOA). Besides, this strengthening of the lower body muscles allows for joint movement, and improves the patient’s functional status [11].

Finally, in a brief report [12] included in this special volume, this author shows the importance of managing polymedication in elderly patients. Some drugs commonly used in older adults produce interactions with cytochrome P450 (CYP), potentially producing pharmacological stress (toxicity) that directly affects the state of health, negatively altering the physical activity capabilities of patients. Thus, the authors [12] suggest a rigorous control of this medication, as it somehow conditions the functional status and quality of life of the elderly.

The twelve manuscripts [1,2,3,4,5,6,7,8,9,10,11,12] published in this Special Issue “*Sarcopenia, Exercise and Quality of Life*” highlight non-pharmacological interventions based on therapeutic physical exercise, with instruments and activities adapted to the older adult population. New diagnostic techniques for sarcopenia are reflected to successfully identify the sarcopenic elderly, and modern descriptors of sarcopenia based on different body composition measurements are established. Medication management is proposed as a critical element in the health status of the older adult.
